# Osmoelectric siphon models for signal and water dispersal in wounded plants

**DOI:** 10.1093/jxb/erac449

**Published:** 2022-11-15

**Authors:** Yong-Qiang Gao, Edward E Farmer

**Affiliations:** Department of Plant Molecular Biology, University of Lausanne, Lausanne, Switzerland; Department of Plant Molecular Biology, University of Lausanne, Lausanne, Switzerland; Nanjing Normal University, China

**Keywords:** DAMP, drought, electrical signal, elicitor, insect, jasmonate, mandibles, PAMP, turgor, water potential

## Abstract

When attacked by herbivores, plants produce electrical signals which can activate the synthesis of the defense mediator jasmonate. These wound-induced membrane potential changes can occur in response to elicitors that are released from damaged plant cells. We list plant-derived elicitors of membrane depolarization. These compounds include the amino acid l-glutamate (Glu), a potential ligand for GLUTAMATE RECEPTOR-LIKE (GLR) proteins that play roles in herbivore-activated electrical signaling. How are membrane depolarization elicitors dispersed in wounded plants? In analogy with widespread turgor-driven cell and organ movements, we propose osmoelectric siphon mechanisms for elicitor transport. These mechanisms are based on membrane depolarization leading to cell water shedding into the apoplast followed by membrane repolarization and water uptake. We discuss two related mechanisms likely to occur in response to small wounds and large wounds that trigger leaf-to-leaf electrical signal propagation. To reduce jasmonate pathway activation, a feeding insect must cut through tissues cleanly. If their mandibles become worn, the herbivore is converted into a robust plant defense activator. Our models may therefore help to explain why numerous plants produce abrasives which can blunt herbivore mouthparts. Finally, if verified, the models we propose may be generalizable for cell to cell transport of water and pathogen-derived regulators.

## Teaser

Fluid carrying elicitors of membrane depolarization is released when cells are damaged. These elicitors trigger water release from nearby cells, providing a mechanism for their transport away from sites of damage.

## Introduction

The vast majority of invertebrate folivores get their food from the largest compartment in the leaf, the symplast. In order to do so, these organisms must first penetrate the leaf’s second largest compartment, the apoplast. Like breaking a window to enter a house, damage to the plasma membrane interface between the apoplast and the symplast can activate alarms. The danger for chewing herbivores is that broken cells release their contents into the apoplast. Some of these molecules act as damage-associated molecular patterns (DAMPs) that elicit immune responses in the host plant (Tanaka and Heil, 2021). Since the perception of these compounds causes defense activation, it is in the interest of the herbivore to minimize their release, propagation, and action. This is especially important for a subset of DAMPs which can elicit the synthesis of the plant defense modulator jasmonoyl-isoleucine (JA-Ile; Howe *et al.*, 2018; J. Wang *et al.*, 2019) and its immediate precursor jasmonic acid (JA). Importantly, in the case of wounding, current evidence suggests that these elicitors act on jasmonate synthesis indirectly by first triggering membrane depolarization. Indeed, a genetic link between wound response membrane potential changes and the activation of JA-Ile synthesis has been established (Mousavi *et al.*, 2013). Here we focus principally on events that take place upstream of wound-induced membrane depolarization.

It is hard to imagine a plant in nature that completes its life cycle without being wounded at least once. Chewing herbivores damage plants in different ways. For example, leaf miners typically tunnel through the mesophyll then emerge as adults by breaking through the epidermis. Lepidopteran caterpillars typically feed from the surfaces of plants. Depending on their developmental stage, they may remove one or a few layers of cells or they may sever large veins. In all cases, chewing herbivores cause mixing of fluids from the symplast and apoplast. The leaf apoplast, comprised of cell walls, extracellular spaces, and xylem vessels, is characterized by a relatively low water potential and, in most cell walls, a surface tension component (McClendon, 1981). Here we suggest that these properties, coupled to the ability of cell membranes to depolarize and repolarize, are likely to be key to plant defense induction by feeding insects.

The majority of this review concentrates on apoplastic dispersal of membrane-depolarizing elicitors derived from the damaged symplasm. In this context, we summarize what is known about the nature of elicitors of membrane depolarization and JA/JA-Ile synthesis. We distinguish short-range events caused by the wounding of one or several cells and long-range (organ to organ) signaling events caused by extensive damage to plant tissues and in particular to veins. The relationship between elicitor dispersion and apoplastic water potential is discussed. We do not cover herbivore- or pathogen-associated molecular patterns (HAMPs and PAMPs) and effectors derived from the herbivores themselves (reviewed in Snoeck *et al.*, 2022). Throughout the review we use the general term ‘elicitor’: the mechanisms we propose could, in theory, transport diverse pathogen- and herbivore-derived molecules. Additionally, we do not discuss cell wall integrity and its connection to jasmonate pathway induction (reviewed in Vaahtera *et al.*, 2019; Wolf, 2022) or touch response electrical signaling; the text is restricted to invasive stimuli.

## Small and large wounds and their relationship to electrical signals

Large and small wounds to aerial tissues can lead to jasmonate pathway activation. For example, small puncture wounds are sufficient to activate jasmonate-dependent defense gene expression in Arabidopsis cotyledons (Acosta *et al.*, 2013). Single cell wounding in Arabidopsis roots causes membrane depolarization which then activates ethylene signaling (Marhavý *et al.*, 2019). In the same study, and although single cell damage-associated electrical signaling in aerial organs was not studied, ablation of single cotyledon cells activated jasmonate signaling. However, membrane depolarization is linked strongly to the activation of jasmonate synthesis (Farmer *et al.*, 2020). Evidence suggests that membrane depolarizations can spread symplastically via plasmodesmata or along the plasma membranes of sieve elements (Fromm and Lautner, 2007; Hedrich *et al.*, 2016). This might occur without the need for chemical modulators of membrane potential. However, other mechanisms to spread changes in membrane potential clearly exist. These involve the chemical elicitation of membrane depolarization by plant-derived elicitors generated or released upon wounding. These compounds can, in theory, be released even from wounds to single cells. Since small wounds trigger localized jasmonate signaling (Acosta *et al.*, 2013; Marhavý *et al.*, 2019), we assume that electrical signals originating from these wounds can travel short distances to nearby undamaged cells. We propose a mechanism that might determine the distance these elicitors travel.

Large wounds inflicted by herbivores inevitably damage veins. Herbivore damage to the primary leaf vasculature triggers leaf-to-leaf electrical signaling. These events are detectable with non-invasive surface electrodes as high-amplitude, long-duration slow-wave potentials (SWPs; Stahlberg *et al.*, 2006). An SWP is characterized by a rapid membrane depolarization phase followed by a slow repolarization phase. Prescient work by Houwink (1935) on the sensitive plant *Mimosa pudica* defined ‘two distinct ways of conduction’; that is, the work hinted at electrical signaling in both the phloem and xylem regions. Indeed, Fromm *et al.* (2013) presented evidence consistent with xylem to phloem electrical signaling in maize. Current research is consistent with this. In Arabidopsis, successful SWP propagation leading to the activation of jasmonate (JA-Ile) synthesis depends on several clade 3 GLUTAMATE RECEPTOR-LIKE (GLR) ion channels that act directly or indirectly as regulators of membrane depolarization. Among these genes are *GLR3.3* and *GLR3.6* (Mousavi *et al.*, 2013). The main cellular localizations of GLR3.3 and GLR3.6 in expanded Arabidopsis leaves support roles for both the phloem and xylem in leaf-to-leaf wound signalling (Nguyen *et al.*, 2018; Toyota *et al.*, 2018). The principal vascular pools of GLR3.3 and GLR3.6 were found in the phloem and xylem, respectively. GLR3.3 protein was also detected in the epidermis (Nguyen *et al.*, 2018). Our knowledge of the subcellular distributions of the GLRs in these cells is, however, still incomplete. At the subcellular level, the major GLR3.3 pools localized to the endoplasmic reticulum in phloem sieve elements. The major GLR3.6 pools were found in the tonoplasts of xylem contact cells. Additional, as yet undiscovered, localizations of these proteins in other vascular and extravascular cells and in other membranes (e.g. the plasma membrane) are likely to exist (Nguyen *et al.*, 2018). Nevertheless, the cellular localizations of the main GLR3.3 and GLR3.6 pools indicate that xylem–phloem interactions must take place during SWP propagation (Nguyen *et al.*, 2018).

## Elicitors of membrane depolarization and jasmonate synthesis

What are the plant-derived elicitors of membrane depolarization that might activate jasmonate production? Table 1 gives a list of membrane-depolarizing elicitors from plants and from a Charophyte alga. The simplest of all these agents is the K^+^ ion. The list includes the reactive oxygen species (ROS), hydrogen peroxide, polycations such as spermine and spermidine, polyanions such as the cell wall component oligogalacturonic acid, as well as DNA and a variety of nucleotides. From the list, it is seen that various sugars and amino acids can trigger membrane potential changes. There are also reports that the hormone indole acetic acid (IAA) can cause membrane depolarization. Table 1 also includes a number of specialized plant metabolites such as phenolic acids and terpenes, as well as several green leaf volatiles. Finally, among the peptides which can trigger membrane depolarization are reduced and oxidized glutathione and also systemin. There are likely to be other peptides that can affect membrane potential leading to jasmonate synthesis. For example, Vega-Muñoz *et al.* (2020) produced a valuable list of molecules including peptides that can induce jasmonate synthesis or signaling. We note that some of these molecules have yet to be tested for their ability to trigger membrane potential changes.

**Table 1.** Examples of plant-derived membrane-depolarizing elicitors

Elicitor	Plant	Tissue/cell	Electrode position	JA response	Reference
K^+^	Arabidopsis	Leaf	Extracellular	NA	Favre *et al.* (2001)
	*Chara corallina*	Internodal cell	Extracellular	NA	Shimmen (2006)
Sucrose	Soybean	Cotyledon	Intracellular	NA	Lichtner and Spanswick (1981)
	Arabidopsis	Seedling	–	JA-Ile content↑	Wingler *et al.* (2020)
Glucose, mannose, galactose	Barley	Leaf	Extracellular	NA	Felle and Zimmermann (2007)
	Liverwort	Thallus cell	Intracellular	NA	Felle and Bentrup (1980)
Amino acids (Glu, Ala, Asn, Cys, Gly, Ser, Arg, etc,) and GABA	Arabidopsis	Root	Intracellular	NA	Qi *et al.* (2006)
		Leaf	Extracellular	NA	Shao *et al.* (2020)
	Barley	Leaf	Extracellular	NA	Felle and Zimmermann (2007)
	Moss	Protonema cell	Intracellular	NA	Koselski *et al.* (2020)
	Arabidopsis	Leaf/seedling	–	JA response transcript induction	Toyota *et al.* (2018); Goto *et al.* (2020)
H_2_O_2_	Lima bean	Leaf	Intracellular	NA	Maffei *et al.* (2006)
	Arabidopsis	Mesophyll cell	Intracellular	NA	Nuhkat *et al.* (2021)
Phenolic acids: salicylic acid, benzoic acid, etc.	Barley	Root epidermal cell	Intracellular	NA	Glass and Dunlop (1974)
	Oat	Coleoptile cell	Intracellular	NA	Bates and Goldsmith (1983)
ABA	Broad bean	Guard cell	Intracellular	NA	Roelfsema *et al.* (2004)
	Arabidopsis	Suspension cell	Intracellular	NA	Brault *et al.* (2004)
	*Salvia miltiorrhiza*	Hairy root	–	MeJA content↑	Yang *et al.* (2012)
IAA	Wheat and maize	Coleoptile	Intracellular	NA	Göring *et al.* (1979)
	Arabidopsis	Root epidermal cell	Intracellular	NA	Dindas *et al.* (2018)
Nucleotides (ATP, GTP, etc.)	Arabidopsis	Root hair	Intracellular	NA	Lew and Dearnaley (2000)
		Seedling	–	JA response transcript induction	Tripathi *et al.* (2018)
Polyamines: spermine, spermidine, etc.	Lima bean	Leaf palisade cells	Intracellular	NA	Ozawa *et al.* (2010)
		Seedling	–	JA content↑	Ozawa *et al.* (2009)
	Pea	Root mature zone cortical cell	Intracellular	NA	Pottosin *et al.* (2014)
GLVs: (E)-2-hexenal, (Z)-3-hexenal, etc.	Tomato	Leaf	Intracellular	NA	Zebelo *et al.* (2012)
Monoterpenes	Cucumber	Root elongating zone	Intracellular	NA	Maffei *et al.* (2001)
esDNA	Lima bean and maize	Leaf	Intracellular	NA	Barbero *et al.* (2016)
Oligogalacturonides	Tomato	Mesophyll cell	Intracellular	NA	Thain *et al.* (1990)
	Tomato	Leaf		JA content↑	Doares *et al.* (1995)
Peptides and proteins					
Systemin	Tomato	Mesophyll cell	Intracellular	NA	Moyen and Johannes (1996)
		Leaf		JA content↑	Doares *et al.* (1995)
GSH and GSSG	Arabidopsis	Root	Intracellular	NA	Qi *et al.*, (2006)
		Leaf		JA response transcript induction	Han *et al.* (2013)
ZmES4 (92 amino acids)	Maize	Pollen tube	Intracellular	NA	Amien *et al.* (2010)
AtPep1/2/3	Arabidopsis	Mesophyll cell	Intracellular	NA	Krol *et al.* (2010)

GLVs, green leaf volatiles; esDNA, extracellular self-DNA; NA, not analysed. Only JA responses induced by exogenously applied elicitors were referred to here.

For the vast majority of the elicitors listed in Table 1, potential links to the activation of jasmonate synthesis or signaling have not been reported. However, several DAMPs that can trigger jasmonate pathway activation are known. These include pectate/pectin-derived cell wall fragments (in particular oligogalacturonides; Doares *et al.*, 1995), the peptide systemin (Moyen and Johannes, 1996), and more recently l-glutamic acid (Glu) which stimulates the expression of jasmonate-response genes (Toyota *et al.*, 2018). A common feature of these diverse compounds is that they all trigger membrane depolarization (Thain *et al.*, 1990; Moyen and Johannes, 1996; Dennison and Spalding, 2000; Shao *et al.*, 2020). We are unaware of reports of the long-distance transport of oligogalacturonides in wounded plants. Concerning systemin, current evidence suggests that this peptide is not necessary for damage-response jasmonate pathway activation in leaves distal to wounds (Wang *et al.*, 2018). However, the amino acid Glu is of special interest since it is a potential ligand for the clade 3 GLR proteins that control membrane depolarization in tissues distal to wounds.

Glu is implicated in diverse signaling processes in plants and has been studied as a potential signal molecule for decades (Forde and Lea, 2007; Qiu *et al.*, 2020; Liao *et al.*, 2022). Interest in the effects of Glu on ion fluxes was prompted by the discovery of *GLR* genes in the Arabidopsis genome (Lam *et al.*, 1998). This helped to stimulate research on the effects of exogenous Glu on membrane potentials in plants. For example, Dennison and Spalding (2000) showed that Glu triggered membrane depolarization in Arabidopsis root tip cells and that this was preceded by a spike-like increase in cytosolic Ca^2+^ levels. Glu was also shown to trigger membrane depolarization in Arabidopsis mesophyll cells (Meyerhoff *et al.*, 2005). Further work showed that like Glu, the amino acids glycine, alanine, asparagine, cysteine, and serine all triggered membrane depolarization in Arabidopsis hypocotyl cells (Stephens *et al.*, 2008). In each case, these amino acids elicited membrane depolarizations which were GLR3.3 dependent. Consistent with this, Glu can bind directly to several clade 3 GLRs. In GLR3.3, Glu is bound in the absence of water, whereas the smaller glycine is bound in the presence of two water molecules (Alfieri *et al.*, 2020). A similar situation pertains for glycine binding to GLR3.2 (Gangwar *et al.*, 2021) and for serine binding to GLR3.4 (Green *et al.*, 2021). Therefore, amino acid binding to clade 3 GLRs might depend to some extent on water potential.

Further interest in Glu as a signaling mediator came from the finding that several clade 3 *GLR* genes underlie electrical signaling in the aerial tissues of wounded Arabidopsis (Mousavi *et al.*, 2013). Toyota *et al.* (2018) revealed that Glu could trigger jasmonate-response gene expression in Arabidopsis leaves. Importantly, the same study provided strong genetic evidence that extracellular Glu levels increase after wounding. Interestingly, the effect of exogenous Glu on membrane depolarization appears to be both concentration and plant specific; that is, one compound, Glu, can elicit at least three types of electrical signals in plants. For example, 1 mM Glu triggered action potentials in barley leaves (Felle and Zimmermann, 2007). However, Zimmermann *et al.* (2009) found that an initial treatment of a barley leaf with 10 mM Glu triggered an action potential, but a second treatment of the leaf with 10 mM Glu triggered a hyperpolarization typical of a system potential. More recently, treatment of the cut ends of Arabidopsis roots or hypocotyls with 50–100 mM Glu was shown to trigger SWP-like membrane depolarizations in Arabidopsis leaves (Shao *et al.*, 2020). How can one compound stimulate the production of electrical signals as diverse as action potentials and SWPs? We suggest that this is due to the fact that Glu may act as an excitotoxin in plants as is known for its effects on animal cells (Ankarcrona *et al.*, 1995). It is possible that, in the constant presence of high Glu levels, cell membranes may remain depolarized for too long, blocking physiological functions. This raises the possibility that other endogenous SWP elicitors might be excitotoxins. Such molecules might act at the cell surface or, for full activity, might need to be imported into cells. If so, their action may lead to particularly efficient water shedding from the elicited cell into the apoplast.

## Elicitor dispersal and the apoplast

**Fig. 1.**
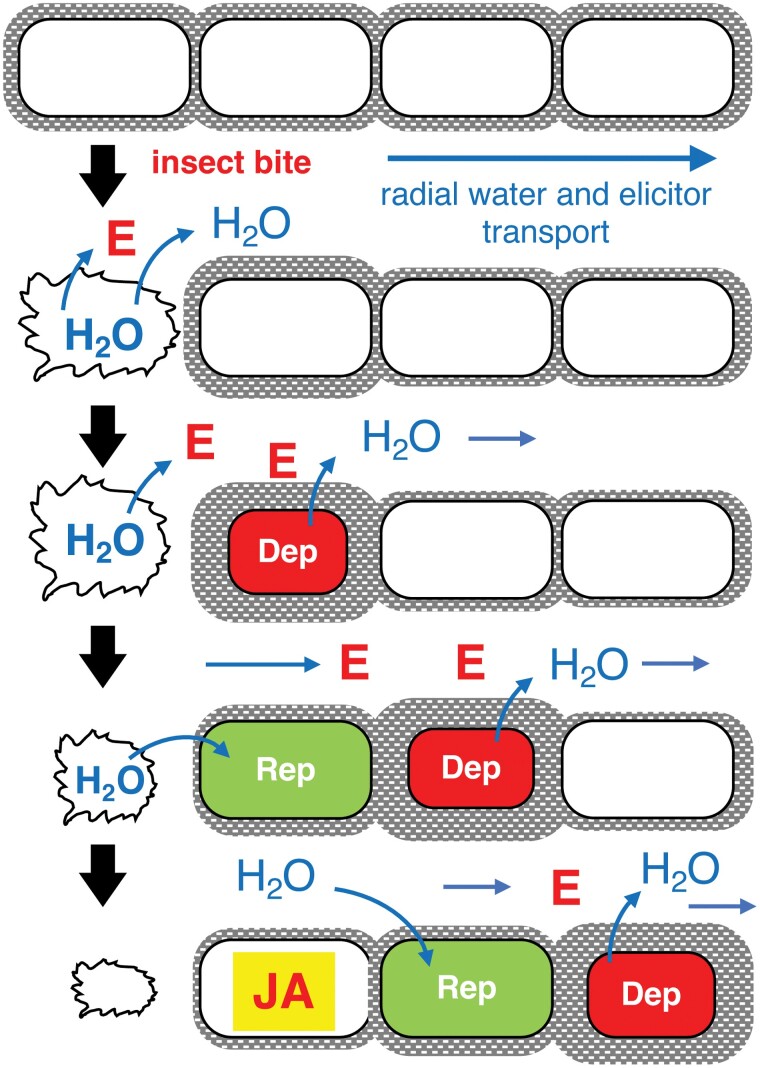
Model for radial elicitor dispersion from a small wound in a cell layer. The fluid contents of broken cells disperse elicitors of membrane depolarization (red Es) to neighboring cells. When these cells (red) depolarize, they lose water. Cell walls adjacent to the wound suck up this water and the elicitors in this fluid depolarize their cells (green), further promoting elicitor distribution in the apoplast. The membranes will then repolarize and their cells will take up water again. In this way, rings of ‘wet’ apoplasm will spread radially from small wounds. After dispersing, the process will terminate when the elicitor concentration falls below a critical level or when the elicitor decays. This process is linked to triggering jasmonate (JA) synthesis. Dep = membrane depolarization; Rep = membrane repolarization.

The thin, shell-like cell walls of the leaf mesophyll have low water potentials in the range of –0.3 MPa (Meinzer and Moore, 1988; Wright and Beattie, 2004). Interestingly, pathogens manipulate the apoplast to increase water potential (Xin *et al.*, 2016; Hernandez and Lindow, 2019). To what extent chewing insects do this is unclear. However, sudden water fluxes into the apoplast caused by herbivore feeding should spread quickly; that is, fluid released from the symplasm of herbivore-damaged cells is expected to ‘wet’ the apoplast and, in doing so, carry with it symplast-derived elicitors of membrane depolarization. This or a similar process is likely to occur when one or a few cells are wounded. Elicitor release from damaged cells would be of little use to plants if the elicitor could not be dispersed effectively. Part of the mechanism of elicitor dispersal will clearly depend on diffusion. For example, the diffusion-driven spread of elicitors such as amino acids through the apoplast is linked tightly to cytosolic Ca^2+^ waves (Bellandi *et al.*, 2022). Here, we propose additional mechanisms which may explain apoplastic elicitor dispersal in wounded plants. The models are based on mechanisms involving tightly associated changes in membrane potential, cell volume, and apoplastic water potential. Insights into the relationships of these processes came, for example, from early work on *Chara corallina*. Experiments with these giant algal cells revealed cell length shortening during membrane depolarization (Oda and Linstead, 1975). Osmoelectric motors involving transmembrane water fluxes underlie the opening and closing of stomata (e.g. Hill and Findlay, 1981; Roelfsema and Hedrich, 2005), a process which can be surprisingly rapid in some ferns (Cardoso *et al.*, 2019). Closely related mechanisms can operate even more rapidly, controlling fast movements associated with prey capture by carnivorous plants (Hill and Findlay, 1981). Also, generally at slower scales, osmotic mechanisms can explain reversible turgor-driven diel movements of leaves and floral organs (Hill and Findlay, 1981) and the cell swelling that underlies plant growth (Forterre, 2013). Here, we add a new aspect; the involvement of elicitor compounds that may drive rapid osmoelectric processes by depolarizing membranes. We herein use the assumption that plant cells in general lose water when their membranes depolarize. Moreover, we predict that osmoelectric siphon mechanisms can help explain elicitor transport in wounded plants. In summary, we propose ‘push–pull’ mechanisms in which cells first shed water into the apoplast when their membranes depolarize upon contact with elicitors. Critically, the same cells can take up water again when their membranes repolarize (Fig. 1).

## A cell-level osmoelectric siphon model for elicitor dispersal from small wounds

A model for apoplastic elicitor dispersal from small wounds (e.g. to one or a few cells in the epidermis or mesophyll) is shown in Fig. 1. The model shows the release of membrane depolarization elicitors from single cell wounds. The essence of the model is that these compounds collapse membrane potentials in surrounding undamaged cells, and that those cells then release water into the apoplasm. The walls of neighboring cells will then attract this water and, in the process, this will carry elicitors further away from the wound. Added to this, and not shown in the model, diffusion will also disperse signal molecules until they are diluted out or they decay. In this way, elicitors from wounds will radiate away from damaged cells. This mechanism depends on low apoplastic water potentials in the undamaged plant. However, apoplastic water potentials are likely to vary depending on environmental conditions. A prediction based on this model is that the lower the apoplastic water potential, the better the spread of elicitors in the apoplast. This may also be the case for large wounds that rupture the vasculature. Such wounds, unlike wounds to the epidermis or mesophyll, cause direct damage to the phloem and xylem.

## Elicitor transport in the xylem in relation to electrical signaling

From the moment that a vein is severed by a feeding insect, and in the seconds that follow, the water potentials of the xylem and phloem must change. Damage to the phloem must lead to some loss of pressure, no matter how fast phloem occlusion occurs. In parallel, fluid from damaged tissues is drawn from the wound site into vessels as their tension is released suddenly (Stahlberg and Cosgrove, 1992). This sap will essentially compete with air to be sucked into the xylem. Importantly, the sap carries with it membrane depolarization elicitors. The spread of the SWP is driven by these elicitors of membrane depolarization as they travel through the xylem of the wounded plant (e.g. Evans and Morris, 2017; Kurenda *et al.*, 2019). This latter point links recent work on Arabidopsis with the historically important proposal for the existence of ‘Ricca’s factors’. These xylem-mobile factors were proposed to control distal wound-response leaf movements in the sensitive plants *Mimosa spegazzinii* and *M. pudica* (Ricca, 1916, 1926). A brief history of Ricca’s factors is given in Box 1.

**Fig. 2.**
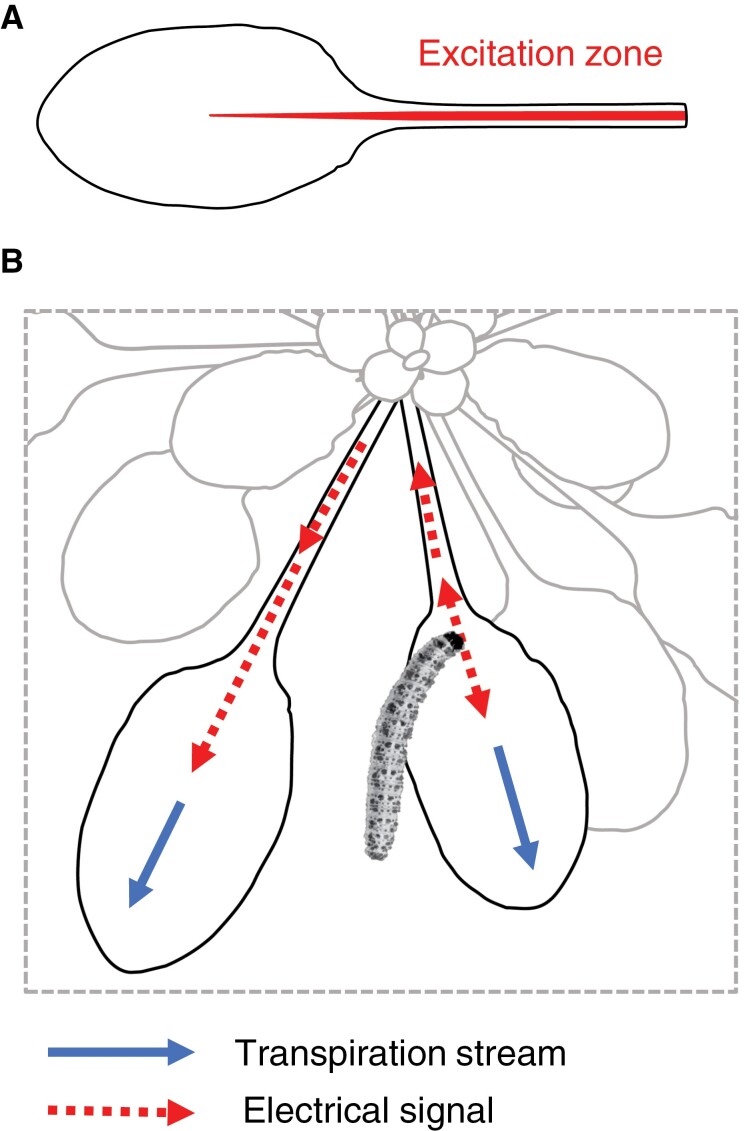
Vein regions important in wound-response electrical signaling. (A) The red highlight (‘excitation zone’) on the Arabidopsis leaf shows regions which must be bitten by insects in order to trigger leaf-to-leaf slow wave potential signaling (Kurenda *et al.*, 2019). (B) The directions of the transpiration streams of two neighboring leaves are indicated with solid blue arrows. Insect damage triggers electrical signaling (dashed red arrows) which travels in the directions indicated.

In order to initiate SWPs that can travel from leaf to leaf, herbivores must damage the basipetal primary veins (Kurenda *et al.*, 2019) in the region illustrated in red in Fig. 2A. Once initiated, SWPs travel to distal leaves that share vascular connections with the wounded leaf (Mousavi *et al.*, 2013). SWPs travel through the primary veins of Arabidopsis at apparent velocities of 6–9 cm min^–1^ (Mousavi *et al.*, 2013; Nguyen *et al.*, 2018). The elicitors of membrane depolarization that are transported from leaf to leaf must travel at similar speeds to these electrical signals. Membrane depolarization in leaves distal to wounds takes place prior to peak cytosolic Ca^2+^ transients (Nguyen *et al.*, 2018). Similarly, peak increases in cytosolic Ca^2+^ levels followed flagellin-induced membrane depolarization in *Nicotiana benthamiana* mesophyll cells (Li *et al.*, 2021).

A likely driver of elicitor transport is transpiration, although other mechanisms such as turbulent diffusion in the xylem have been proposed (Vodeneev *et al.*, 2012). In the case of Arabidopsis, and as illustrated in Fig. 2B, electrical signals travel basipetally from sites of damage to the primary vein and then disperse into leaves that share direct vascular connections with the damaged leaf. We note that, in the damaged leaf, electrical signals can travel towards the plant center. One explanation for this is that all leaves essentially compete for water supplied by the root. When the petiolar primary vein of one leaf is severed, leaves sharing vascular connections with this leaf outcompete its reduced transpiration, reversing xylem water flow in the severed petiole. This might help to explain the fact that the apparent velocity of the SWP in Arabidopsis is faster in the distal unwounded leaf than in the wounded leaf (Mousavi *et al.*, 2013).

Here we consider transpiration as a primary driver of SWP elicitor transport. SWP generation is extremely robust in wild-type plants. When intact wild-type Arabidopsis plants were placed in the dark for 3 d and then transferred to the light, the plants displayed typical SWPs (Fotouhi *et al.*, 2022). In the same study, caterpillar-damaged wild-type plants that lacked much if not most of their laminar tissues were still capable of propagating SWPs. In this case, the SWP propagation velocity was 2-fold slower in these damaged leaves relative to leaves with intact laminas. While transpiration appears to play an important role in xylem elicitor dispersion, careful studies of the link between transpiration and SWP propagation hint at additional mechanisms involved in elicitor transport. For example, Stahlberg *et al.* (2005) concluded that transpiration rate was unlikely to be the only determinant of SWP propagation in sunflower leaves. Furthermore, SWP signaling needs to function at different times of the day and under different soil water potentials. For this, a mechanism related to that proposed for short-distance signaling in Fig. 1 may act together with transpiration-driven elicitor transport.

## An osmoelectric siphon model for elicitor dispersal in the vasculature

**Fig. 3.**
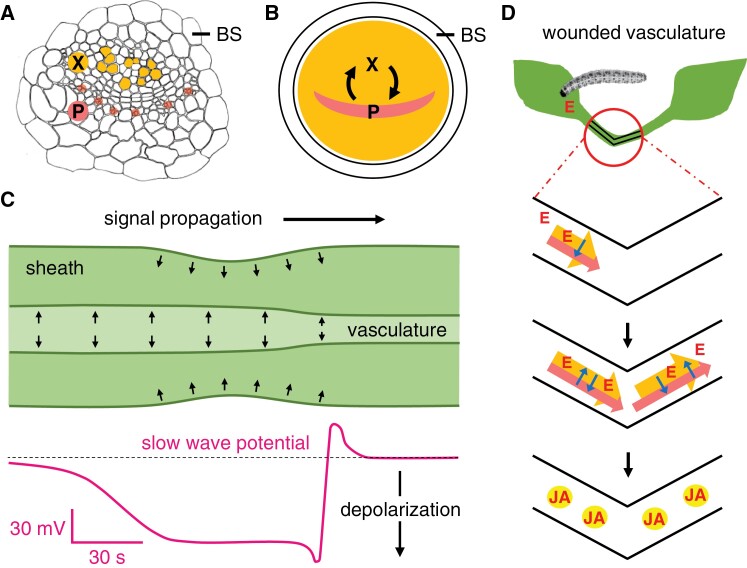
Two-field model for electrical signaling and elicitor dispersion leading to jasmonate synthesis. (A) Transversal view of a primary vein from an Arabidopsis leaf. BS, bundle sheath; X, xylem; P, phloem. Xylem vessels are colored orange in the image. Phloem sieve elements and companion cells are distributed in poles indicated as pink dots. (B) Hypothetical fields of cells involved in electrical signaling. One field (orange) is panvascular and the other field (pink) is in the region of the phloem (P). It is of note that the phloem and xylem fields are separated by a cambial layer. The possible consequences of electrical signaling across this developmentally important tissue have not, to our knowledge, been explored. (C) Putative pressure increases within the vascular bundle and petiole surface deformation in relation to SWP electrical activity (based on results from Kurenda *et al.*, 2019). (D) Wounding triggers depolarization of the panvascular field (orange) and the phloem field (pink). Elicitors (red Es, otherwise termed ‘Ricca’s factors’) from damaged cells spread along xylem vessels and depolarize cells in the xylem field. Membrane potential changes in the two fields of cells interact (blue arrows). Elicitor movement in the xylem allows the SWP to travel from the wounded leaf to distal leaves.

The relative positions of the xylem and phloem in an Arabidopsis primary vein are shown in Fig. 3A. In healthy plants including trees, the xylem and phloem operate together under diverse conditions (e.g. Spicer, 2014; Stroock *et al.*, 2014; Van Bel, 1990; Zwieniecki *et al.*, 2004; J. Knoblauch *et al.*, 2016; Konrad *et al.*, 2019). We assume that this is also the case in the wounded plant and that there is a coordinated function of the phloem and xylem in leaf-to-leaf electrical signaling. Figure 3B illustrates the positions of fields of cells associated with the xylem and phloem. The phloem field includes sieve elements, companion cells, and the many associated cells which are usually referred to as phloem parenchyma. The phloem electrical field is embedded in a panvascular electrical field which includes that of xylem contact cells. From an electrophysiology perspective, we assume that cells in these two fields can be depolarized in a coordinated manner upon attack. Herein, we envisage that the vasculature, and in particular the phloem, acts as a kind of capacitor (Fotouhi *et al.*, 2022). In this view, the ability of the phloem to discharge (depolarize) and repolarize rapidly and fully is vital for the electrical activities of other vascular cells.

Both cell rupture and simply compressing cells without breaking them may trigger electrical events. Regarding non-damaging pressure changes, previous work led to the hypothesis that compressive forces on xylem contact cells are associated with SWP signaling (Farmer *et al.*, 2014). Here, based on recent experimental observations, we extend this ‘squeeze cell’ hypothesis to larger fields of vascular cells. Primary veins in Arabidopsis leaves swell as wound-response electrical signals travel from leaf to leaf (Kurenda *et al.*, 2019). In parallel, deformations of the petiole surfaces were detected (Fig. 3C). Both of these observations are consistent with water fluxes in the tissues of wounded plants. We speculate that increased axial water fluxes through vessels in damaged plants may be coupled to radial water shedding from the xylem into nearby cell walls.

In Fig. 3D we envisage an electrical field associated with the phloem. Other vascular cells, including those in the xylem region, form a second theoretical field. We speculate that these fields interact and operate together in wound-response electrical signaling. How this occurs is unknown. However, certain features of the cell matrix that comprises the Arabidopsis vasculature may provide clues. The Arabidopsis primary vein (Fig. 3A), unlike the mesophyll, lacks intercellular air spaces. This may facilitate extensive radial interactions between vascular cells via plasmodesmata; such interactions may be important for wound-response electrical signaling. To function effectively in defense as activators of jasmonate synthesis, electrical signals need to be propagated axially to distal leaves. This is facilitated by xylem-borne membrane depolarization elicitors (‘Ricca’s factors’) that essentially ‘bridge the gap’ between leaves.

**Fig. 4.**
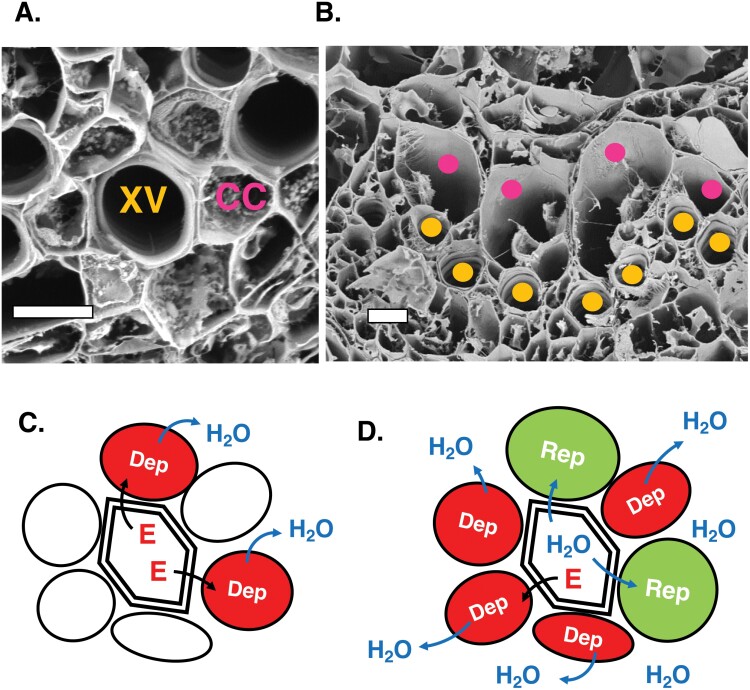
An osmoelectric siphon model for drawing water and elicitors into the vascular apoplasm in response to wounding. (A) Cryo-fracture electron microscopy image of an array of contact cells surrounding a vessel in a primary vein from an Arabidopsis petiole. The image was from a region roughly midway between the petiole base and the lamina. Orange XV, xylem vessel; purple CC, contact cell. Scale bar=10 μm. (B) Enlarged contact cells (purple dots) at the base of an Arabidopsis petiole. These cells are of potential interest with respect to osmoelectrical models and possibly for diel leaf movements. Brown dots are placed on several xylem vessels. Scale bar=10 μm. (C) Model for a mechanism to draw water and elicitors along xylem vessels and into the apoplasm in a wounded plant. Following damage to the vasculature, elicitors (red E) are released into xylem vessels and drawn along the transpiration stream. These elicitors depolarize contact cell membranes which then shed water. Cells with depolarized membranes are indicated in red. (D) Upon repolarization, water from vessels is preferentially reloaded from the xylem into contact cells. This mechanism is envisaged to operate in wounded leaves and to allow elicitor transport to undamaged leaves distal to wounds. Repolarizing cells are indicated in green. The model assumes that there is a largely one-way flow of water from vessels to contact cells. Jasmonate synthesis is not indicated. For (A) and (B) images were prepared using cryo-fracture electron microscopy of leaves and petioles from 4.5-week-old Arabidopsis plants. Dep = membrane depolarization; Rep = membrane repolarization.

Figure 2B illustrates that in order to elicit distal defense signaling, elicitors from damaged leaves must be transported basipetally from the damaged leaf before they can enter the transpiration stream of an undamaged leaf. Plants that have been badly damaged by herbivores may have a reduced capacity for transpiration. However, such plants still produce SWPs, albeit of slower velocities than those of intact plants (Fotouhi *et al.*, 2022). In cases like these, we speculate that osmoelectric siphons may help to facilitate basipetal elicitor transport in damaged leaves so that elicitors can be channeled towards transpiration streams in leaves distal to wounds. Xylem contact cells are often found as stellate arrangements organized around vessels. Such a cluster from the mid-region of an Arabidopsis petiole is shown in Fig. 4A. However, depending on their position in the primary vein, these elongated cells can have variable diameters. At the base of the Arabidopsis petiole, some contact cells that are adaxially positioned relative to vessels can be greatly enlarged (Fig. 4B). These petiole–base contact cells should, in theory, have a large capacity to shed water. In the model, the membranes of vessel-associated cells in damaged leaves come into contact with plant-derived elicitors. These cells then depolarize and lose water which is drawn away into the apoplast (Fig. 4C). As the contact cells repolarize, they preferentially take up water from vessels, thereby pulling water and elicitors along the xylem (Fig. 4D).

The main assumption of the model is that xylem contact cells and other core vascular cells release water when their membranes depolarize. Much of this water may originate in vacuoles. Indeed, changes in vacuole sizes have already been linked strongly to electrical signaling. For example, electrical signals in *M. pudica* trigger rapid pulvinar turgor changes, water movements, and changes in vacuole size and shape (e.g. Sibaoka, 1991). Could similar but more cryptic events take place in Arabidopsis? Intriguingly, the vacuoles of Arabidopsis xylem contact cells show remarkably variable volumes depending on whether or not vein samples are prepared in the presence or absence of sorbitol (Nguyen *et al.*, 2018). It will therefore be of interest to investigate contact cell vacuole dynamics upon wounding. A further aspect that needs testing is the possibility that the velocity and/or extent of leaf-to-leaf electrical signaling will depend in part on leaf water potentials.

**Box 1. Ricca’s factors in a nutshell**

For decades, the touch- and wound-induced movements of *Mimosa pudica* have intrigued biologists. This plant (along with related species such as *M. spegazzinii*) became ‘the’ model for touch-response signaling and for leaf-to-leaf wound signaling. These plants attracted the attention of physiologists including Henri Dutrochet, Wilhelm Pfeffer, and Gottlieb Haberlandt. Each of these scientists and some of their forerunners pondered possible mechanisms of signal propagation within touch-stimulated or damaged *Mimosa*. Building on their work, Ricca (1916, 1926) showed that factors present in extracts from *M. pudica* leaves could travel through dead tissues or even through glass tubes to elicit distal leaf movements in this plant. This led to a sustained but ultimately unsuccessful effort to isolate these factors (e.g. Fitting, 1930, 1936; Van Sambeek *et al.*, 1976). Meanwhile, Houwink (1935) made the connection between leaf movements in *M. pudica* and electrical signals in this plant. Houwink’s work supported Ricca’s proposal that what are now called Ricca’s factors (RFs) travel from wounds through the xylem to distal leaves. Moreover, Houwink specifically associated RFs to damage-induced membrane depolarizations that he called ‘variations’ and that are now known as either ‘variation potentials’ or ‘slow wave potentials’ (SWPs; as used herein). It is noteworthy that RFs were proposed to elicit the long-duration depolarization phase of the SWP (Houwink, 1935).

For years, the RF concept remained *Mimosa* specific. However, Barbara Pickard broadened the definition of RFs to xylem-mobile electrical signal-inducing substances from plants (Van Sambeek *et al.*, 1976). At this point, the generality of the RF concept became increasingly clear—all plants should have RFs. However, the roles of these putative substances in plants other than *M. pudica* remained unclear. Pickard evoked a possible role for SWPs in plant defense (Van Sambeek *et al.*, 1976). This later turned out to be the case when electrical signals produced in response to wounding were genetically linked to the activation of the synthesis of jasmonate in Arabidopsis (Mousavi *et al.*, 2013). The current perspective is that xylem-mobile substances underlie wound-response leaf-to-leaf electrical signaling in Arabidopsis (Evans and Morris, 2017). Indeed, evidence supports a role for xylem-mobile RFs in electrical signaling leading to both minute leaf movements in wounded Arabidopsis and jasmonate signaling induction in this plant (Kurenda *et al.*, 2019). GLR proteins appear to act downstream of RFs to control leaf movements and jasmonate pathway activation in Arabidopsis. What, then, is the chemical nature of RFs? Are they common and conserved molecules or are they plant specific? Sibaoka (1997) carried out reciprocal treatments of different plants with leaf extracts and, failing to find strong evidence for interspecific RF action, concluded that that these molecules might be ‘species specific’.

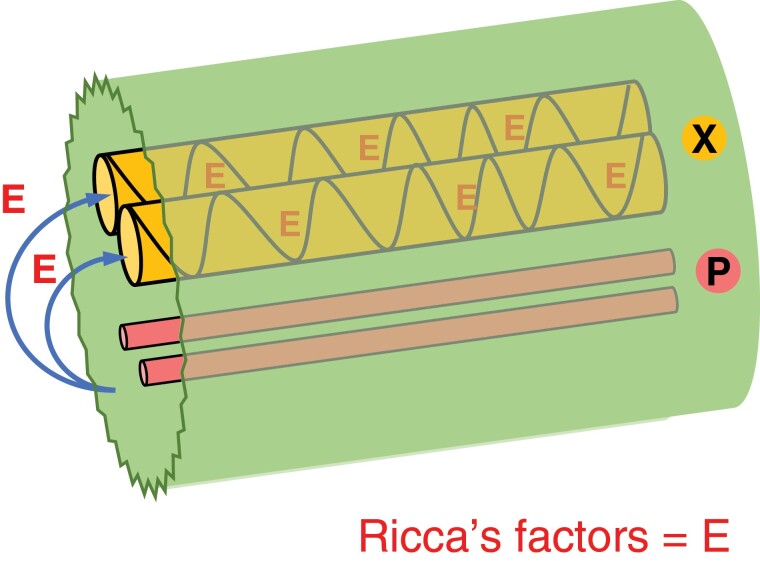

A vein showing the regions of the xylem (X) and phloem (P) within a broken vein (green). Elicitors (E) are released from vascular issues and are drawn into the xylem. The compounds are also known as Ricca’s factors after classic work by Ubaldo Ricca (1916, 1926).

## Relationship of osmomelectric siphon models and mechanohydraulic models

Our osmoelectric models differ from current mechanohydraulic models used in studies of cell growth (e.g. Long *et al.*, 2020). Mechanohydraulic models are generally used to explore irreversible cell growth trajectories. The osmoelectric models herein are based on reversible transmembrane charge redistribution and water fluxes leading to reversible (elastic) changes in cell volume. We note that cells adjacent to wounds divide in the hours that follow wounding (Hoermayer *et al.*, 2020). The osmoelectric mechanisms proposed herein should work over shorter time frames (tens of seconds) and their relationship to cell division, if any, is yet to be explored. Although mechanohydraulic and osmoelectric models have different applications, they are united by hydraulics. Therefore, interactions between the two types of model are possible.

## Testing osmolectric siphon models

Following genetic tests to ensure the biological relevance of a given elicitor, the spatial extent of elicitation output (e.g. jasmonate signaling and electrical signaling) could be established with reporter genes and, where possible, surface electrodes. Even more challenging tests will involve measuring the degree of water shedding from elicited cells. This may be problematic in part due to the fact that cell-specific apoplastic water potentials (matric potentials), apoplastic solute compositions, and conductivities and ionic strengths are not yet measurable using non-damaging methods in intact plants. Elicited cells should lose turgor and this could be measured, for example with atomic force microscopy. Force sensors such as those employed in Kurenda *et al.* (2019) may also be useful at the tissue level. A potential caveat is that water influx into the apoplast might, in addition to affecting turgor, alter cell wall properties, for example causing wall swelling similar to that observed in brown algae (M. Knoblauch *et al.*, 2016). That possibility would need to be taken into account. Pico gauges (Knoblauch *et al.*, 2014) could be used to probe intracellular pressures. However, the possibility that puncturing cells might itself trigger membrane depolarization will need consideration. Osmolyte treatments may be considered. For example, the above wild-type level of jasmonate signaling observed in Arabidopsis *korrigan1* (*kor1*) cell wall mutants was suppressed by hyperosmotic treatments (Mielke *et al.*, 2021). However, we note that in the absence of membrane depolarization elicitors, osmolytes alone may not be sufficient to strongly depolarize the cell membranes. Moreover, key to the models we present are apoplastic water fluxes, and these may be reduced or abolished if cells are partially dehydrated in hyperosmotic conditions.

## The elicitation of jasmonate synthesis after wounding

Xylem contact cells are sites of wound-response jasmonate precursor synthesis in leaves distal to wounds (Chauvin *et al.*, 2013; Gasperini *et al.*, 2015). Jasmonate precursor synthesis in these cells follows SWP signaling; however, the key regulatory steps that allow the initiation of jasmonate synthesis in these cells are unknown. More is known about the synthesis of jasmonates during reproductive development. Developmental jasmonate production in flowers requires lipases (Ishiguro *et al.*, 2001), and these enzymes are also known to play roles in jasmonate synthesis initiation in wounded leaves (Ellinger *et al.*, 2010). The activity of such lipases may depend on wound-response increases in cytosolic Ca^2+^ which probably regulate post-wounding jasmonate synthesis (X. Wang *et al.*, 2019). Three other factors might affect jasmonate synthesis in contact cells. Each of these is related to the SWP: membrane depolarization, changes in water potential, and changes in intra/intercellular pressures. Genetic approaches have shown that intracellular pressures can trigger jasmonate responses (Mielke *et al.*, 2021). Also concerning pressure, we note that mechanosensitive ion channels are expressed in plastids which can deform in their absence (Haswell and Meyerowitz, 2006). Alternatively, it is possible that plasma membrane potential changes and/or changes in water potential might affect endomembranes including those of plastids. Such mechanisms could, in theory, trigger galactolipid phase changes which then allow lipases to generate jasmonate precursors. The potential contributions of membrane depolarization, rapid cell water potential changes, and tissue pressure changes to the wound-activated initiation of jasmonate synthesis need investigation. In any case, feeding herbivores need to minimize the activation of jasmonate synthesis.

## Potential relevance in biotic and abiotic stress

The mandibles of herbivorous insects have forms that are adapted to their host plants (Bernays, 1991). Chewing insects generally cut plant tissues using sharp mandibles, avoiding crushing of cells surrounding the sites they feed on. There are exceptions to this. For example, some insect larvae tear leaf tissues (Bernays and Janzen, 1988) and some lepidopterans use their mandibles to generate sounds (Brown *et al.*, 2007). However, all these herbivores have developed feeding strategies that allow the rapid procurement and digestion of food, thereby ensuring their fast growth (Bernays and Janzen, 1988). This is facilitated by having sharp mandibles. Here, we propose another potential effect of mandible wear—this time on the plant itself.

If osmoelectric siphons function in plant defense, the degree to which a feeding insect activates the jasmonate pathway will depend in part on its mandibles. As a widespread anti-herbivore defense mechanism, plants commonly deploy vitreous, silicon-based abrasives (Debona *et al.*, 2017; Coskun *et al.*, 2019; Johnson *et al.*, 2021) or crystalline deposits such as calcium oxalate (Nakata, 2003) that can blunt insect mandibles (Korth *et al.*, 2006; Massey and Hartley, 2009; Park *et al.*, 2009). This is thought to reduce the ability of herbivores to digest plant tissues efficiently, therefore retarding herbivore development (Bernays, 1991). In some cases, defense chemicals generated upon wounding are thought to occlude the mouthparts of insects that feed by sucking rather than chewing (Bai *et al.*, 2022). What are the consequences of insect mandible/mouthpart blunting for the plant? For example, is it the same to be clipped by razor-sharp mouthparts or by mandibles that have been blunted and worn? Based on our models for elicitor dispersal, we propose that mandible wear will affect plant defense response induction. With relevance to the feeding mode of the herbivore, it is likely that, when vascular tissues are crushed rather than severed cleanly, elicitor-rich fluids enter the xylem more efficiently than does air. Clearly, the herbivore must minimize the release of symplast-derived elicitors of membrane depolarization into the apoplast. Herbivores can do so by severing tissues with sharp mandibles. Finally, the models presented could be of interest in terms of drought stress. For example, the model in Fig. 1 emphasizes the fact that a dying cell sheds its water into the apoplast. Under severe drought stress, living cells will start to lose their membrane potentials and will shed water. Nearby cells will attract and gather this water from the dying cell, thereby minimizing rapid evaporative water loss from the tissue.

## Conclusion

We present models for the transport of water, elicitors, and effectors in herbivore- (or pathogen) attacked plants. In the models we develop, chemical elicitors trigger membrane potential changes and water fluxes that are associated with the activation of the jasmonate pathway. These osmoelectric siphon models involve elicitor-induced exchange of water between the symplast and the apoplast correlated with changes in membrane potential. At more negative membrane potentials, cell turgor is maintained; however, turgor is lost when membrane potentials collapse. We speculate that electrical signaling in attacked plants may, in addition to other mechanisms, exploit the fluid continuum of the apoplast to actively disperse elicitors. This would allow plants to couple and coordinate membrane depolarizations within and between cell layers. A prediction is that the more turgid a cell is, the greater its response to elicitation. Throughout the text, we assume that the same elicitor triggers both membrane depolarization and subsequent water shedding. However, the possibility that multiple elicitors are involved in one of these two processes needs consideration.

For small (non-vascular) wounds, we propose a mechanism in which elicitors may be dispersed efficiently over short distances in the apoplasm so that even small quantities of these molecules are carried away from the wound site. We distinguish this first model from a second model for elicitor dispersion within the vasculature of severely wounded tissues. Leaf-to-leaf electrical signaling must be robust enough to function day and night and under constantly varying environmental conditions. To explain this robustness, mechanisms going beyond transpiration have been evoked herein. This second model involves the xylem and the phloem. Decades of research indicate that the phloem is of vital importance in axial electrical signaling through veins. Phloem electrical signals triggered by non-damaging stimuli are generally restricted to the stimulated organ and these signals can be relatively fast depending on the plant species (Fromm and Lautner, 2007). It is not yet known whether phloem electrical signaling in response to non-damaging stimuli requires phloem-borne chemical mediators of membrane potential change. However, severe wounding of aerial tissues triggers leaf-to-leaf electrical signaling in a process that clearly requires the long-distance transport of membrane depolarization elicitors through the xylem. Viewed simply, the phloem in the wounded plant offers a fast route for electrical signaling while the xylem offers a slower route. Elicitors which travel through the xylem are likely to stimulate electrical signaling in the phloem. That is, in the presence of plant-derived xylem-borne elicitors, the phloem electrical signal which would otherwise be restricted to the damaged leaf can overcome an effective barrier to reach distal leaves. If verified, the models developed herein may also help to explain the movement of herbivore- and pathogen-derived effectors. In the future, it will be interesting to verify the relationships (if any) between osmoelectric siphon models and mechanohydraulic models. Lastly, some aspects of the models we present may be relevant to the study of cell-to-cell water transport in drought-stressed plants.

## References

[CIT0001] Acosta IF , Gasperini D, Chételat A, Stolz S, Santuari L, Farmer EE. 2013. Role of NINJA in root jasmonate signaling. Proceedings of the National Academy of Sciences, USA 110, 15473–15478. 10.1073/pnas.1307910110 PMC3780868 24003128

[CIT0002] Alfieri A , Doccula FG, Pederzoli R, Grenzi M, Bonza MC, Luoni L, Candeo A, Armada NR, Barbiroli A, Valentini G. 2020. The structural bases for agonist diversity in an *Arabidopsis thaliana* glutamate receptor-like channel. Proceedings of the National Academy of Sciences, USA 117, 752–760. 10.1073/pnas.1905142117 PMC6955363 31871183

[CIT0003] Amien S , Kliwer I, Márton ML, Debener T, Geiger D, Becker D, Dresselhaus T. 2010. Defensin-like ZmES4 mediates pollen tube burst in maize via opening of the potassium channel KZM1. PLoS Biology 8, e1000388. 20532241 10.1371/journal.pbio.1000388 PMC2879413

[CIT0004] Ankarcrona M , Dypbukt JM, Bonfoco E, Zhivotovsky B, Orrenius S, Lipton SA, Nicotera P. 1995. Glutamate-induced neuronal death: a succession of necrosis or apoptosis depending on mitochondrial function. Neuron 15, 961–973. 7576644 10.1016/0896-6273(95)90186-8

[CIT0005] Bai Y , Yang C, Halitschke R, Paetz C, Kessler D, Burkard K, Gaquerel E, Baldwin IT, Li D. 2022. Natural history-guided omics reveals plant defensive chemistry against leafhopper pests. Science 375, eabm2948. 35113706 10.1126/science.abm2948

[CIT0006] Barbero F , Guglielmotto M, Capuzzo A, Maffei ME. 2016. Extracellular self-DNA esDNA, but not heterologous plant or insect DNA etDNA, induces plasma membrane depolarization and calcium signaling in Lima bean *Phaseolus lunatus* and maize *Zea mays*. International Journal of Molecular Sciences 17, 1659. 27690017 10.3390/ijms17101659 PMC5085692

[CIT0007] Bates GW , Goldsmith MHM. 1983. Rapid response of the plasma-membrane potential in oat coleoptiles to auxin and other weak acids. Planta 159, 231–237. 24258173 10.1007/BF00397530

[CIT0008] Bellandi A , Papp D, Breakspear A, et al. 2022. Diffusion and bulk flow of amino acids mediate calcium waves in plants. Science Advances 8, eabo6693. 36269836 10.1126/sciadv.abo6693 PMC9586480

[CIT0009] Bernays EA. 1991. Evolution of insect morphology in relation to plants. Philosophical Transactions of the Royal Society B: Biological Sciences 333, 257–264.

[CIT0010] Bernays E , Janzen DH. 1988. Saturniid and sphingid caterpillars: two ways to eat leaves. Ecology 69, 1153–1160.

[CIT0011] Brault M , Amiar Z, Pennarun AM, Monestiez M, Zhang Z, Cornel D, Dellis O, Knight H, Bouteau F, Rona JP. 2004. Plasma membrane depolarization induced by abscisic acid in *Arabidopsis* suspension cells involves reduction of proton pumping in addition to anion channel activation, which are both Ca^2+^ dependent. Plant Physiology 135, 231–243. 15141069 10.1104/pp.104.039255 PMC429360

[CIT0012] Brown SG , Boettner GH, Yack JE. 2007. Clicking caterpillars: acoustic aposematism in *Antheraea polyphemus* and other Bombycoidea. Journal of Experimental Biology 210, 993–1005. 17337712 10.1242/jeb.001990

[CIT0013] Cardoso AA , Randall JM, McAdam SA. 2019. Hydraulics regulate stomatal responses to changes in leaf water status in the fern *Athyrium filix-femina*. Plant Physiology 179, 533–543. 30538169 10.1104/pp.18.01412 PMC6426430

[CIT0014] Chauvin A , Caldelari D, Wolfender JL, Farmer EE. 2013. Four 13-lipoxygenases contribute to rapid jasmonate synthesis in wounded *Arabidopsis thaliana* leaves: a role for lipoxygenase 6 in responses to long‐distance wound signals. New Phytologist 197, 566–575. 23171345 10.1111/nph.12029

[CIT0015] Coskun D , Deshmukh R, Sonah H, Menzies JG, Reynolds O, Ma JF, Kronzucker HJ, Bélanger RR. 2019. The controversies of silicon’s role in plant biology. New Phytologist 221, 67–85. 30007071 10.1111/nph.15343

[CIT0016] Debona D , Rodrigues FA, Datnoff LE. 2017. Silicon’s role in abiotic and biotic plant stresses. Annual Review of Phytopathology 55, 85–107. 10.1146/annurev-phyto-080516-035312 28504920

[CIT0017] Dennison KL , Spalding EP. 2000. Glutamate-gated calcium fluxes in *Arabidopsis*. Plant Physiology 124, 1511–1514. 11115867 10.1104/pp.124.4.1511 PMC1539305

[CIT0018] Dindas J , Scherzer S, Roelfsema MRG, von Meyer K, Müller HM, Al-Rasheid K, Palme K, Dietrich P, Becker D, Bennett MJ. 2018. AUX1-mediated root hair auxin influx governs SCFTIR1/AFB-type Ca^2+^ signaling. Nature Communications 9, 1–10. 10.1038/s41467-018-03582-5 PMC5862985 29563504

[CIT0019] Doares SH , Syrovets T, Weiler EW, Ryan CA. 1995. Oligogalacturonides and chitosan activate plant defensive genes through the octadecanoid pathway. Proceedings of the National Academy of Sciences, USA 92, 4095–4098. 10.1073/pnas.92.10.4095 PMC41892 11607534

[CIT0020] Ellinger D , Stingl N, Kubigsteltig II, Bals T, Juenger M, Pollmann S, Berger S, Schuenemann D, Mueller MJ. 2010. DONGLE and DEFECTIVE IN ANTHER DEHISCENCE1 lipases are not essential for wound- and pathogen-induced jasmonate biosynthesis: redundant lipases contribute to jasmonate formation. Plant Physiology 153, 114–127. 20348210 10.1104/pp.110.155093 PMC2862439

[CIT0021] Evans MJ , Morris RJ. 2017. Chemical agents transported by xylem mass flow propagate variation potentials. The Plant Journal 91, 1029–1037. 28656705 10.1111/tpj.13624 PMC5601289

[CIT0022] Farmer EE , Gao YQ, Lenzoni G, Wolfender JL, Wu Q. 2020. Wound- and mechanostimulated electrical signals control hormone responses. New Phytologist 227, 1037–1050. 32392391 10.1111/nph.16646

[CIT0023] Farmer EE , Gasperini D, Acosta IF. 2014. The squeeze cell hypothesis for the activation of jasmonate synthesis in response to wounding. New Phytologist 204, 282–288. 25453132 10.1111/nph.12897

[CIT0024] Favre P , Greppin H, Degli Agosti R. 2001. Repetitive action potentials induced in *Arabidopsis thaliana* leaves by wounding and potassium chloride application. Plant Physiology and Biochemistry 39, 961–969.

[CIT0025] Felle H , Bentrup FW. 1980. Hexose transport and membrane depolarization in *Riccia fluitans*. Planta 147, 471–476. 24311171 10.1007/BF00380190

[CIT0026] Felle HH , Zimmermann MR. 2007. Systemic signalling in barley through action potentials. Planta 226, 203–214. 17226028 10.1007/s00425-006-0458-y

[CIT0027] Fitting H. 1930. Untersuchungen uber endogene Chemonastie bei *Mimosa pudica*. Jahrbücher für wissenschaftliche Botanik 72, 700–775.

[CIT0028] Fitting H. 1936. Untersuchungen über die chemischen Eigenschaften des Reizstoffes von *Mimosa pudica*. Jahrbücher für Wissenschaftliche Botanik 83, 270–314.

[CIT0029] Forde BG , Lea PJ. 2007. Glutamate in plants: metabolism, regulation, and signalling. Journal of Experimental Botany 58, 2339–2358. 17578865 10.1093/jxb/erm121

[CIT0030] Forterre Y. 2013. Slow, fast and furious: understanding the physics of plant movements. Journal of Experimental Botany 64, 4745–4760. 23913956 10.1093/jxb/ert230

[CIT0031] Fotouhi N , Fischer-Stettler M, Lenzoni G, Stolz S, Glauser G, Zeeman SC, Farmer EE. 2022. ACA pumps maintain leaf excitability during herbivore onslaught. Current Biology 32, 2517–2528.e6.e6 2517. 10.1016/j.cub.2022.03.059 35413240

[CIT0032] Fromm J , Hajirezaei MR, Becker VK, Lautner S. 2013. Electrical signaling along the phloem and its physiological responses in the maize leaf. Frontiers in Plant Science 4, 239. 23847642 10.3389/fpls.2013.00239 PMC3701874

[CIT0033] Fromm J , Lautner S. 2007. Electrical signals and their physiological significance in plants. Plant, Cell & Environment 30, 249–257. 10.1111/j.1365-3040.2006.01614.x 17263772

[CIT0034] Gangwar SP , Green MN, Michard E, Simon AA, Feijo JA, Sobolevsky AI. 2021. Structure of the *Arabidopsis* glutamate receptor-like channel GLR3.2 ligand-binding domain. Structure 29, 161–169. 10.1016/j.str.2020.09.006 PMC7867599 33027636

[CIT0035] Gasperini D , Chauvin A, Acosta IF, Kurenda A, Stolz S, Chételat A, Wolfender JL, Farmer EE. 2015. Axial and radial oxylipin transport. Plant Physiology 169, 2244–2254. 26338953 10.1104/pp.15.01104 PMC4634084

[CIT0036] Glass AD , Dunlop J. 1974. Influence of phenolic acids on ion uptake: IV. Depolarization of membrane potentials. Plant Physiology 54, 855–858. 16658989 10.1104/pp.54.6.855 PMC366622

[CIT0037] Göring H , Polevoy V, Stahlberg R, Stumpe G. 1979. Depolarization of transmembrane potential of corn and wheat coleoptiles under reduced water potential and after IAA application. Plant & Cell Physiology 20, 649–656.

[CIT0038] Goto Y , Maki N, Ichihashi Y, Kitazawa D, Igarashi D, Kadota Y, Shirasu K. 2020. Exogenous treatment with glutamate induces immune responses in *Arabidopsis*. Molecular Plant-Microbe Interactions 33, 474–487. 31721650 10.1094/MPMI-09-19-0262-R

[CIT0039] Green MN , Gangwar SP, Michard E, Simon AA, Portes MT, Barbosa-Caro J, Wudick MM, Lizzio MA, Klykov O, Yelshanskaya MV. 2021. Structure of the *Arabidopsis thaliana* glutamate receptor-like channel GLR3.4. Molecular Cell 81, 3216–3226. 10.1016/j.molcel.2021.05.025 PMC8349882 34161757

[CIT0040] Han Y , Mhamdi A, Chaouch S, Noctor G. 2013. Regulation of basal and oxidative stress-triggered jasmonic acid-related gene expression by glutathione. Plant, Cell & Environment 36, 1135–1146. 10.1111/pce.12048 23210597

[CIT0041] Haswell ES , Meyerowitz EM. 2006. MscS-like proteins control plastid size and shape in *Arabidopsis thaliana*. Current Biology 16, 1–11. 16401419 10.1016/j.cub.2005.11.044

[CIT0042] Hedrich R , Salvador-Recatalà V, Dreyer I. 2016. Electrical wiring and long-distance plant communication. Trends in Plant Science 21, 376–387. 26880317 10.1016/j.tplants.2016.01.016

[CIT0043] Hernandez MN , Lindow SE. 2019. *Pseudomonas syringae* increases water availability in leaf microenvironments via production of hygroscopic syringafactin. Applied and Environmental Microbiology 85, e01014–e01019. 31285194 10.1128/AEM.01014-19 PMC6715840

[CIT0044] Hill BS , Findlay GP. 1981. The power of movement in plants: the role of osmotic machines. Quarterly Reviews of Biophysics 14, 173–222. 7025083 10.1017/s0033583500002249

[CIT0045] Hoermayer L , Montesinos JC, Marhava P, Benková E, Yoshida S, Friml J. 2020. Wounding-induced changes in cellular pressure and localized auxin signalling spatially coordinate restorative divisions in roots. Proceedings of the National Academy of Sciences, USA 117, 15322–15331. 10.1073/pnas.2003346117 PMC7334516 32541049

[CIT0046] Houwink AL. 1935. The conduction of excitation in *Mimosa pudica*. Recueil Des Travaux Botaniques Neerlandais 32, 51–91.

[CIT0047] Howe GA , Major IT, Koo AJ. 2018. Modularity in jasmonate signaling for multistress resilience. Annual Review of Plant Biology 69, 387–415. 10.1146/annurev-arplant-042817-040047 29539269

[CIT0048] Ishiguro S , Kawai-Oda A, Ueda J, Nishida I, Okada K. 2001. The DEFECTIVE IN ANTHER DEHISCENCE1 gene encodes a novel phospholipase A1 catalyzing the initial step of jasmonic acid biosynthesis, which synchronizes pollen maturation, anther dehiscence, and flower opening in *Arabidopsis*. The Plant Cell 13, 2191–2209. 11595796 10.1105/tpc.010192 PMC139153

[CIT0049] Johnson SN , Hartley SE, Ryalls JM, Frew A, Hall CR. 2021. Targeted plant defense: silicon conserves hormonal defense signaling impacting chewing but not fluid-feeding herbivores. Ecology 102, e03250. 33219513 10.1002/ecy.3250

[CIT0050] Knoblauch J , Mullendore DL, Jensen KH, Knoblauch M. 2014. Pico gauges for minimally invasive intracellular hydrostatic pressure measurements. Plant Physiology 166, 1271–1279. 25232014 10.1104/pp.114.245746 PMC4226368

[CIT0051] Knoblauch J , Tepler Drobnitch S, Peters WS, Knoblauch M. 2016. *In situ* microscopy reveals reversible cell wall swelling in kelp sieve tubes: one mechanism for turgor generation and flow control? Plant, Cell & Environment 39, 1727–1736. 10.1111/pce.12736 26991892

[CIT0052] Knoblauch M , Knoblauch J, Mullendore DL, Savage JA, Babst BA, Beecher SD, Dodgen AC, Jensen KH, Holbrook NM. 2016. Testing the Münch hypothesis of long distance phloem transport in plants. eLife 5, e15341. 27253062 10.7554/eLife.15341 PMC4946904

[CIT0053] Konrad W , Katul G, Roth-Nebelsick A, Jensen KH. 2019. Xylem functioning, dysfunction and repair: a physical perspective and implications for phloem transport. Tree Physiology 39, 243–261. 30299503 10.1093/treephys/tpy097

[CIT0054] Korth KL , Doege SJ, Park SH, Goggin FL, Wang Q, Gomez SK, Liu G, Jia L, Nakata PA. 2006. *Medicago truncatula* mutants demonstrate the role of plant calcium oxalate crystals as an effective defense against chewing insects. Plant Physiology 141, 188–195. 16514014 10.1104/pp.106.076737 PMC1459329

[CIT0055] Koselski M , Wasko P, Derylo K, Tchorzewski M, Trebacz K. 2020. Glutamate-induced electrical and calcium signals in the moss *Physcomitrella patens*. Plant & Cell Physiology 61, 1807–1817. 32810281 10.1093/pcp/pcaa109

[CIT0056] Krol E , Mentzel T, Chinchilla D, et al. 2010. Perception of the *Arabidopsis* danger signal peptide 1 involves the pattern recognition receptor AtPEPR1 and its close homologue AtPEPR2. Journal of Biological Chemistry 285, 13471–13479. 20200150 10.1074/jbc.M109.097394 PMC2859507

[CIT0057] Kurenda A , Nguyen CT, Chételat A, Stolz S, Farmer EE. 2019. Insect-damaged *Arabidopsis* moves like wounded *Mimosa pudica*. Proceedings of the National Academy of Sciences, USA 116, 26066–26071. 10.1073/pnas.1912386116 PMC6926025 31792188

[CIT0058] Lam HM , Chiu J, Hsieh MH, Meisel L, Oliveira IC, Shin M, Coruzzi G. 1998. Glutamate-receptor genes in plants. Nature 396, 125–126. 9823891 10.1038/24066

[CIT0059] Lew RR , Dearnaley JD. 2000. Extracellular nucleotide effects on the electrical properties of growing *Arabidopsis thaliana* root hairs. Plant Science 153, 1–6.

[CIT0060] Li K , Prada J, Damineli DS, Liese A, Romeis T, Dandekar T, Feijó JA, Hedrich R, Konrad KR. 2021. An optimized genetically encoded dual reporter for simultaneous ratio imaging of Ca^2+^ and H^+^ reveals new insights into ion signaling in plants. New Phytologist 230, 2292–2310. 33455006 10.1111/nph.17202 PMC8383442

[CIT0061] Liao HS , Chung YH, Hsieh MH. 2022. Glutamate: a multifunctional amino acid in plants. Plant Science 318, 111238. 35351313 10.1016/j.plantsci.2022.111238

[CIT0062] Lichtner FT , Spanswick RM. 1981. Electrogenic sucrose transport in developing soybean cotyledons. Plant Physiology 67, 869–874. 16661771 10.1104/pp.67.4.869 PMC425789

[CIT0063] Long Y , Cheddadi I, Mosca G, Mirabet V, Dumond M, Kiss A, Traas J, Godin C, Boudaoud A. 2020. Cellular heterogeneity in pressure and growth emerges from tissue topology and geometry. Current Biology 30, 1504–1516.e8. 32169211 10.1016/j.cub.2020.02.027

[CIT0064] Maffei M , Camusso W, Sacco S. 2001. Effect of *Mentha* × *piperita* essential oil and monoterpenes on cucumber root membrane potential. Phytochemistry 58, 703–707. 11672734 10.1016/s0031-9422(01)00313-2

[CIT0065] Maffei ME , Mithöfer A, Arimura GI, Uchtenhagen H, Bossi S, Bertea CM, Cucuzza LS, Novero M, Volpe V, Quadro S. 2006. Effects of feeding *Spodoptera littoralis* on lima bean leaves. III. Membrane depolarization and involvement of hydrogen peroxide. Plant Physiology 140, 1022–1035. 16443697 10.1104/pp.105.071993 PMC1400574

[CIT0066] Marhavý P , Kurenda A, Siddique S, Dénervaud Tendon V, Zhou F, Holbein J, Hasan MS, Grundler FM, Farmer EE, Geldner N. 2019. Single-cell damage elicits regional, nematode‐restricting ethylene responses in roots. The EMBO Journal 38, e100972. 31061171 10.15252/embj.2018100972 PMC6518030

[CIT0067] Massey FP , Hartley SE. 2009. Physical defences wear you down: progressive and irreversible impacts of silica on insect herbivores. Journal of Animal Ecology 78, 281–291. 18771503 10.1111/j.1365-2656.2008.01472.x

[CIT0068] McClendon JH. 1981. The balance of forces generated by the water potential in the cell-wall-matrix—a model. American Journal of Botany 68, 1263–1268.

[CIT0069] Meinzer FC , Moore PH. 1988. Effect of apoplastic solutes on water potential in elongating sugarcane leaves. Plant Physiology 86, 873–879. 16666002 10.1104/pp.86.3.873 PMC1054586

[CIT0070] Meyerhoff O , Müller K, Roelfsema MRG, Latz A, Lacombe B, Hedrich R, Dietrich P, Becker D. 2005. AtGLR3.4, a glutamate receptor channel-like gene is sensitive to touch and cold. Planta 222, 418–427. 15864638 10.1007/s00425-005-1551-3

[CIT0071] Mielke S , Zimmer M, Meena MK, Dreos R, Stellmach H, Hause B, Voiniciuc C, Gasperini D. 2021. Jasmonate biosynthesis arising from altered cell walls is prompted by turgor-driven mechanical compression. Science Advances 7, eabf0356. 33568489 10.1126/sciadv.abf0356 PMC7875531

[CIT0072] Mousavi SA , Chauvin A, Pascaud F, Kellenberger S, Farmer EE. 2013. *GLUTAMATE RECEPTOR-LIKE* genes mediate leaf-to-leaf wound signalling. Nature 500, 422–426. 23969459 10.1038/nature12478

[CIT0073] Moyen C , Johannes E. 1996. Systemin transiently depolarizes the tomato mesophyll cell membrane and antagonizes fusicoccin-induced extracellular acidification of mesophyll tissue. Plant, Cell & Environment 19, 464–470.

[CIT0074] Nakata PA. 2003. Advances in our understanding of calcium oxalate crystal formation and function in plants. Plant Science 164, 901–909.

[CIT0075] Nguyen CT , Kurenda A, Stolz S, Chételat A, Farmer EE. 2018. Identification of cell populations necessary for leaf-to-leaf electrical signaling in a wounded plant. Proceedings of the National Academy of Sciences, USA 115, 10178–10183. 10.1073/pnas.1807049115 PMC6176584 30228123

[CIT0076] Nuhkat M , Brosche M, Stoelzle-Feix S, Dietrich P, Hedrich R, Roelfsema MRG, Kollist H. 2021. Rapid depolarization and cytosolic calcium increase go hand-in-hand in mesophyll cells’ ozone response. New Phytologist 232, 1692–1702. 34482538 10.1111/nph.17711

[CIT0077] Oda K , Linstead PJ. 1975. Changes in cell length during action potentials in *Chara*. Journal of Experimental Botany, 26, 228–239.

[CIT0078] Ozawa R , Bertea CM, Foti M, Narayana R, Arimura GI, Muroi A, Horiuchi JI, Nishioka T, Maffei ME, Takabayashi J. 2009. Exogenous polyamines elicit herbivore-induced volatiles in lima bean leaves: involvement of calcium, H_2_O_2_ and jasmonic acid. Plant & Cell Physiology 50, 2183–2199. 19884250 10.1093/pcp/pcp153

[CIT0079] Ozawa R , Bertea CM, Foti M, Ravishankar Narayana R, Arimura GI, Muroi A, Maffei ME, Takabayashi J. 2010. Polyamines and jasmonic acid induce plasma membrane potential variations in Lima bean. Plant Signaling & Behavior 5, 308–310. 20200489 10.4161/psb.5.3.10848 PMC2881286

[CIT0080] Park SH , Doege SJ, Nakata PA, Korth KL. 2009. *Medicago truncatula*‐derived calcium oxalate crystals have a negative impact on chewing insect performance via their physical properties. Entomologia Experimentalis et Applicata 131, 208–215.

[CIT0081] Pottosin I , Velarde-Buendía AM, Bose J, Fuglsang AT, Shabala S. 2014. Polyamines cause plasma membrane depolarization, activate Ca^2+^-, and modulate H^+^-ATPase pump activity in pea roots. Journal of Experimental Botany 65, 2463–2472. 24723394 10.1093/jxb/eru133

[CIT0082] Qi Z , Stephens NR, Spalding EP. 2006. Calcium entry mediated by GLR3.3, an *Arabidopsis* glutamate receptor with a broad agonist profile. Plant Physiology 142, 963–971. 17012403 10.1104/pp.106.088989 PMC1630757

[CIT0083] Qiu XM , Sun YY, Ye XY, Li ZG. 2020. Signaling role of glutamate in plants. Frontiers in Plant Science 10, 1743. 32063909 10.3389/fpls.2019.01743 PMC6999156

[CIT0084] Ricca U. 1916. Soluzione d’un problema di fisologia. La propagazione di stimulo nella *Mimosa*. Nuovo Giornale Botanico Italiano 23, 51–170.

[CIT0085] Ricca U. 1926. Transmission of stimuli in plants. Nature 117, 654–655.

[CIT0086] Roelfsema MRG , Hedrich R. 2005. In the light of stomatal opening: new insights into ‘the Watergate’. New Phytologist 167, 665–691. 16101906 10.1111/j.1469-8137.2005.01460.x

[CIT0087] Roelfsema MRG , Levchenko V, Hedrich R. 2004. ABA depolarizes guard cells in intact plants, through a transient activation of R- and S-type anion channels. The Plant Journal 37, 578–588. 14756768 10.1111/j.1365-313x.2003.01985.x

[CIT0088] Shao Q , Gao Q, Lhamo D, Zhang H, Luan S. 2020. Two glutamate- and pH-regulated Ca^2+^ channels are required for systemic wound signaling in *Arabidopsis*. Science Signaling 13, eaba1453. 32665412 10.1126/scisignal.aba1453

[CIT0089] Shimmen T. 2006. Electrical perception of the ‘death message’ in Chara: characterization of K^+^-induced depolarization. Plant & Cell Physiology 47, 559–562. 16473860 10.1093/pcp/pcj018

[CIT0090] Sibaoka T. 1991. Rapid plant movements triggered by action potentials. The Botanical Magazine 104, 73–95.

[CIT0091] Sibaoka T. 1997. Application of leaf extract causes repetitive action potentials in *Biophytum sensitivum*. Journal of Plant Research 110, 485–487.

[CIT0092] Snoeck S , Guayazán-Palacios N, Steinbrenner AD. 2022. Molecular tug-of-war: plant immune recognition of herbivory. The Plant Cell 34, 1497–1513. 35026025 10.1093/plcell/koac009 PMC9048929

[CIT0093] Spicer R. 2014. Symplasmic networks in secondary vascular tissues: parenchyma distribution and activity supporting long-distance transport. Journal of Experimental Botany 65, 1829–1848. 24453225 10.1093/jxb/ert459

[CIT0094] Stahlberg R , Cleland RE, Volkenburgh EV. 2005. Decrement and amplification of slow wave potentials during their propagation in *Helianthus annuus L.* shoots. Planta 220, 550–558. 15365838 10.1007/s00425-004-1363-x

[CIT0095] Stahlberg R , Cleland RE, Volkenburgh EV. 2006. Slow wave potentials—a propagating electrical signal unique to higher plants. In: Baluška F, Mancuso S, Volkmann D, eds. Communication in plants. Berlin: Springer, 291–308.

[CIT0096] Stahlberg R , Cosgrove DJ. 1992. Rapid alterations in growth rate and electrical potentials upon stem excision in pea seedlings. Planta 187, 523–531. 11538115 10.1007/BF00199972

[CIT0097] Stephens NR , Qi Z, Spalding EP. 2008. Glutamate receptor subtypes evidenced by differences in desensitization and dependence on the *GLR3.3* and *GLR3.4* genes. Plant Physiology 146, 529–538. 18162597 10.1104/pp.107.108134 PMC2245834

[CIT0098] Stroock AD , Pagay VV, Zwieniecki MA, Michele Holbrook N. 2014. The physicochemical hydrodynamics of vascular plants. Annual Review of Fluid Mechanics 46, 615–642.

[CIT0099] Tanaka K , Heil M. 2021. Damage-associated molecular patterns DAMPs in plant innate immunity: applying the danger model and evolutionary perspectives. Annual Review of Phytopathology 59, 53–75. 10.1146/annurev-phyto-082718-100146 33900789

[CIT0100] Thain J , Doherty H, Bowles D, Wildon D. 1990. Oligosaccharides that induce proteinase inhibitor activity in tomato plants cause depolarization of tomato leaf cells. Plant, Cell & Environment 13, 569–574.

[CIT0101] Toyota M , Spencer D, Sawai-Toyota S, Jiaqi W, Zhang T, Koo AJ, Howe GA, Gilroy S. 2018. Glutamate triggers long-distance, calcium-based plant defense signaling. Science 361, 1112–1115. 30213912 10.1126/science.aat7744

[CIT0102] Tripathi D , Zhang T, Koo AJ, Stacey G, Tanaka K. 2018. Extracellular ATP acts on jasmonate signaling to reinforce plant defense. Plant Physiology 176, 511–523. 29180381 10.1104/pp.17.01477 PMC6108377

[CIT0103] Vaahtera L , Schulz J, Hamann T. 2019. Cell wall integrity maintenance during plant development and interaction with the environment. Nature Plants 5, 924–932. 31506641 10.1038/s41477-019-0502-0

[CIT0104] Van Bel AJE. 1990. Xylem–phloem exchange via the rays: the undervalued route of transport. Journal of Experimental Botany 41, 631–644.

[CIT0105] Van Sambeek JW , Pickard BG, Ulbright CE. 1976. Mediation of rapid electrical, metabolic, transpirational, and photosynthetic changes by factors released from wounds. II. Mediation of the variation potential by Ricca’s factor. Canadian Journal of Botany 54, 2651–2661.

[CIT0106] Vega-Muñoz I , Duran-Flores D, Fernández-Fernández AD, Heyman J, Ritter A, Stael S. 2020. Breaking bad news: dynamic molecular mechanisms of wound response in plants. Frontiers in Plant Science 11, 1959. 10.3389/fpls.2020.610445 PMC7752953 33363562

[CIT0107] Vodeneev V , Orlova A, Morozova E, Orlova L, Akinchits E, Orlova O, Sukhov V. 2012. The mechanism of propagation of variation potentials in wheat leaves. Journal of Plant Physiology 169, 949–954. 22533926 10.1016/j.jplph.2012.02.013

[CIT0108] Wang J , Wu D, Wang Y, Xie D. 2019. Jasmonate action in plant defense against insects. Journal of Experimental Botany 70, 3391–3400. 30976791 10.1093/jxb/erz174

[CIT0109] Wang L , Einig E, Almeida-Trapp M, Albert M, Fliegmann J, Mithöfer A, Kalbacher H, Felix G. 2018. The systemin receptor SYR1 enhances resistance of tomato against herbivorous insects. Nature Plants 4, 152–156. 29459726 10.1038/s41477-018-0106-0

[CIT0110] Wang X , Zhu B, Jiang Z, Wang S. 2019. Calcium-mediation of jasmonate biosynthesis and signaling in plants. Plant Science 287, 110192. 31481228 10.1016/j.plantsci.2019.110192

[CIT0111] Wingler A , Tijero V, Müller M, Yuan B, Munné-Bosch S. 2020. Interactions between sucrose and jasmonate signalling in the response to cold stress. BMC Plant Biology 20, 176. 32321430 10.1186/s12870-020-02376-6 PMC7178619

[CIT0112] Wolf S. 2022. Cell wall signaling in plant development and defense. Annual Review of Plant Biology 73, 323–353. 10.1146/annurev-arplant-102820-095312 35167757

[CIT0113] Wright CA , Beattie GA. 2004. *Pseudomonas syringae* pv. *tomato* cells encounter inhibitory levels of water stress during the hypersensitive response of *Arabidopsis thaliana*. Proceedings of the National Academy of Sciences, USA 101, 3269–3274. 10.1073/pnas.0400461101 PMC365779 14981249

[CIT0114] Xin XF , Nomura K, Aung K, Velásquez AC, Yao J, Boutrot F, Chang JH, Zipfel C, He SY. 2016. Bacteria establish an aqueous living space in plants crucial for virulence. Nature 539, 524–529. 27882964 10.1038/nature20166 PMC5135018

[CIT0115] Yang D , Ma P, Liang X, Wei Z, Liang Z, Liu Y, Liu F. 2012. PEG and ABA trigger methyl jasmonate accumulation to induce the MEP pathway and increase tanshinone production in *Salvia miltiorrhiza* hairy roots. Physiologia Plantarum 146, 173–183. 22356467 10.1111/j.1399-3054.2012.01603.x

[CIT0116] Zebelo SA , Matsui K, Ozawa R, Maffei ME. 2012. Plasma membrane potential depolarization and cytosolic calcium flux are early events involved in tomato *Solanum lycopersicon* plant-to-plant communication. Plant Science 196, 93–100. 23017903 10.1016/j.plantsci.2012.08.006

[CIT0117] Zimmermann MR , Maischak H, Mithöfer A, Boland W, Felle HH. 2009. System potentials, a novel electrical long-distance apoplastic signal in plants, induced by wounding. Plant Physiology 149, 1593–1600. 19129416 10.1104/pp.108.133884 PMC2649404

[CIT0118] Zwieniecki MA , Melcher PJ, Feild TS, Holbrook NM. 2004. A potential role for xylem–phloem interactions in the hydraulic architecture of trees: effects of phloem girdling on xylem hydraulic conductance. Tree Physiology 24, 911–917. 15172841 10.1093/treephys/24.8.911

